# Author Correction: Fractional quantum ferroelectricity

**DOI:** 10.1038/s41467-024-53618-2

**Published:** 2024-10-29

**Authors:** Junyi Ji, Guoliang Yu, Changsong Xu, H. J. Xiang

**Affiliations:** 1 Key Laboratory of Computational Physical Sciences (Ministry of Education), Institute of Computational Physical Sciences, State Key Laboratory of Surface Physics, and Department of Physics, Fudan University, Shanghai, 200433 China; 2 Shanghai Qi Zhi Institute, Shanghai, 200030 China; 3 Collaborative Innovation Center of Advanced Microstructures, Nanjing, 210093 China

Correction to: *Nature Communications* 10.1038/s41467-023-44453-y, published online 02 January 2024

The original version of this Article contained an error in Abstract, which incorrectly read ‘28 potential point groups.’ The correct version states ’27 potential point groups’ in place of ’28 potential point groups.’

The main text originally incorrectly read ’28 point groups, of which 8 are polar.’ The correct version states ‘’27 point groups, of which 7 are polar’ instead of ‘’28 point groups, of which 8 are polar’.

The main text originally incorrectly read ’28 possible *P*_L_, while do not include *C*_1_, *C*_6v_, *D*_6h_, *O*_h_ point groups, see Table S1. Interestingly, among the 28 possible *P*_L_, 8 *P*_L_ are polar point groups.’ The correct version states ‘’27 possible *P*_L_, while do not include *C*_1_, *C*_6_, *C*_6v_, *D*_6h_, *O*_h_ point groups, see Table S1. Interestingly, among the 27 possible *P*_L_, 7 *P*_L_ are polar point groups’.

The main text originally incorrectly read ’28 point groups.’ The correct version states ‘’27 point groups’.

This has been corrected in both the PDF and HTML versions of the Article.

The original version of the Supplementary Information associated with this Article contained an error in Table S1, which incorrectly read ‘24 abcd C2 2.’ The correct version states ‘24 abc C2 2’ in place of 24 abcd C2 2’.

The correct version states ‘88 cd Ci 2’ in place of ‘88 cd Ci 4’.

The correct version states ‘115 g C2v 2’ in place of ‘115 g C2 2’.

The correct version states ‘125 ab D4 2’ in place of ‘125 ab D2 2’.

The correct version states ‘140 k Cs 2’ in place of ‘140 kl Cs 2’.

The correct version states ‘150 d C3 3’ in place of ‘150 d C3 2’.

The correct version states ‘179 ab C2 6’ in place of ‘179 ab C3 6’.

The correct version states ‘182 gh C2 2’ in place of ‘182 h C2 2’.

The correct version states ‘188 k Cs 2’ in place of ‘188 kl Cs 2’.

The correct version states ‘194 b D3h 2’ in place of ‘194 b D3h 3’.

The correct version states ‘201 bc C3i 2’ in place of ‘201 bc D3d 2’.

The correct version states ‘203 cd C3i 4’ in place of ‘203 cd D3d 4’.

The correct version states ‘204 c C3i 2’ in place of ‘204 c D3d 2’.

The correct version states ‘205 ab C3i 2’ in place of ‘205 ab D3d 2’.

The correct version states ‘206 ab C3i 2’ in place of ‘206 ab D3d 2’.

The correct version states ‘212 ab D3 4’ in place of ‘212 ab T 4’.

The correct version states ‘213 ab D3 4’ in place of ‘213 ab T 4’.

The correct version states ‘214 ab D3 4’ in place of ‘214 ab T 4’.

The correct version states ‘214 cd D2 8’ in place of ‘214 cd D2 4’.

The correct version states ‘222 c C3i 2’ in place of ‘222 c D3d 2’.

The correct version states ‘222 e C4 2’ in place of ‘222 ef C4 2’.

The correct version states ‘223 a Th 2’ in place of ‘223 a m-3 2’.

The correct version states ‘229 c D3d 2’ in place of ‘229 c D3h 4’.

The HTML has been updated to include a corrected version of the Supplementary Information.

## Supplementary information

Updated Supplementary Information

