# Supplementary material for: Fractional quantum ferroelectricity
Source: Nat Commun. 2024 Jan 2;15:135. doi: 10.1038/s41467-023-44453-y (PMC10761868; doi:10.1038/s41467-023-44453-y)
Supplement: Supplementary file 1 — Supplementary Information [file 41467_2023_44453_MOESM1_ESM.pdf]

Fractional Quantum Ferroelectricity

Junyi Ji[1,2], Guoliang Yu[1,2], Changsong Xu[1,2]*, and H. J. Xiang[1,2,3]*

[1]*Key Laboratory of Computational Physical Sciences (Ministry of Education), Institute of Computational Physical Sciences, State Key Laboratory of Surface Physics, and Department of Physics, Fudan University, Shanghai 200433, China*

[2]*Shanghai Qi Zhi Institute, Shanghai 200030, China*

[3]*Collaborative Innovation Center of Advanced Microstructures, Nanjing 210093, China*

[†]*J.J. and G.Y. contributed equally to this work.*

Email: csxu@fudan.edu.cn, hxiang@fudan.edu.cn

CONTENTS

1. G_F-P_L Pairs of FQFE 1

2. The FQFE in Sc_2CO_2 16

3. The Wyckoff Position Tables of *P-3m1* and *Fm-3m* 17

4. Materials with both *F-43m* and *Fm-3m* phases 20

5. Ferroelasticity of non-polar FQFE 21

6. The thermal-stability analysis of FQFE materials 22

7. FQFE in organic-inorganic materials 23

8. Energy barrier and polarization difference along different switching paths for monolayer α-In_2Se_3. 24

This supplementary material provides further information of fractional quantum ferroelectricity (FQFE).

1. G_F-P_L Pairs of FQFE

A structure L_1 (belong to space group G_L) that has FQFE can be divided into two parts, i.e. F and M. The space group of F, G_F, is the supergroup of G_L. The Wyckoff position of each atom M^i in G_F should be one listed in Tab. S1.

Table. S1. All the possible G_F-P_L pairs that may exhibit FQFE with one mobile M atom. The site symmetry group of the Wyckoff position (letter) in G_F is P_L, the point group of the low symmetry phase. n is the denominator of the fraction in $\frac{m}{n}\mathbf{Q}$. $\frac{m}{n}$ should be one of $\left\{\frac{1}{2},\frac{1}{3},\frac{2}{3},\frac{1}{4},\frac{3}{4},\frac{1}{6},\frac{5}{6},\frac{1}{8},\frac{3}{8},\frac{5}{8},\frac{7}{8}\right\}$ in the conventional cell. $\mathbf{Q}$ is the polarization quantum.

The Index of Space Group G_F	Wyckoff Position (Letter)	Point Group P_L	n of $\frac{m}{n}\mathbf{Q}$
1	\	\	\
2	\	\	\
3	\	\	\
4	\	\	\
5	\	\	\
6	\	\	\
7	\	\	\
8	\	\	\
9	\	\	\
10	\	\	\
11	abcd	*Ci*	2
11	e	*Cs*	2
12	ef	*Ci*	2
13	abcd	*Ci*	2
13	ef	*C2*	2
14	abcd	*Ci*	2
15	abcd	*Ci*	2
15	e	*C2*	2
16	\	\	\
17	abcd	*C2*	2
18	ab	*C2*	2
19	\	\	\
20	ab	*C2*	2
21	k	*C2*	2
22	hij	*C2*	2
23	\	\	\
24	abc	*C2*	2
25	\	\	\
26	\	\	\
27	\	\	\
28	ab	*C2*	2
28	c	*Cs*	2
29	\	\	\
30	ab	*C2*	2
31	a	*Cs*	2
32	ab	*C2*	2
33	\	\	\
34	ab	*C2*	2
35	c	*C2*	2
36	\	\	\

37	c	*C2*	2
38	\	\	\
39	ab	*C2*	2
39	c	*Cs*	2
40	a	*C2*	2
40	b	*Cs*	2
41	a	*C2*	2
42	b	*C2*	2
43	a	*C2*	4
44	\	\	\
45	ab	*C2*	2
46	a	*C2*	2
46	b	*Cs*	2
47	\	\	\
48	abcd	*D2*	2
48	ef	*Ci*	2
48	ghijkl	*C2*	2
49	abcd	*C2h*	2
49	efgh	*D2*	2
49	ijkl	*C2*	2
49	q	*Cs*	2
50	abcd	*D2*	2
50	ef	*Ci*	2
50	ghijkl	*C2*	2
51	abcd	*C2h*	2
51	ef	*C2v*	2
51	gh	*C2*	2
51	k	*Cs*	2
52	ab	*Ci*	2
52	cd	*C2*	2
53	abcd	*C2h*	2
53	efg	*C2*	2
53	h	*Cs*	2
54	ab	*Ci*	2
54	cde	*C2*	2
55	abcd	*C2h*	2
55	ef	*C2*	2
56	ab	*Ci*	2
56	cd	*C2*	2
57	ab	*Ci*	2
57	c	*C2*	2
57	d	*Cs*	2
58	abcd	*C2h*	2

58	ef	*C2*	2
58	g	*Cs*	2
59	ab	*C2v*	2
59	cd	*Ci*	2
59	ef	*Cs*	2
60	ab	*Ci*	2
60	c	*C2*	2
61	ab	*Ci*	2
62	ab	*Ci*	2
62	c	*Cs*	2
63	ab	*C2h*	2
63	c	*C2v*	2
63	d	*Ci*	2
63	e	*C2*	2
63	g	*Cs*	2
64	ab	*C2h*	2
64	c	*Ci*	2
64	de	*C2*	2
65	ef	*C2h*	2
65	m	*C2*	2
66	ab	*D2*	2
66	cdef	*C2h*	2
66	ghk	*C2*	2
66	l	*Cs*	2
67	ab	*D2*	2
67	cdef	*C2h*	2
67	g	*C2v*	2
67	hijkl	*C2*	2
67	n	*Cs*	2
68	ab	*D2*	2
68	cd	*Ci*	2
68	efgh	*C2*	2
69	cde	*C2h*	2
69	f	*D2*	2
69	jkl	*C2*	2
70	ab	*D2*	4
70	cd	*Ci*	4
70	efg	*C2*	4
71	k	*Ci*	2
72	ab	*D2*	2
72	cd	*C2h*	2
72	e	*Ci*	2
72	fghi	*C2*	2

73	ab	*Ci*	2
73	cde	*C2*	2
74	abcd	*C2h*	2
74	e	*C2v*	2
74	fg	*C2*	2
74	i	*Cs*	2
75	c	*C2*	2
76	\	\	\
77	c	*C2*	2
78	\	\	\
79	b	*C2*	2
80	a	*C2*	2
81	g	*C2*	2
82	f	*C2*	2
83	ef	*C2h*	2
83	i	*C2*	2
84	abcd	*C2h*	2
84	ef	*S4*	2
84	i	*C2*	2
84	j	*Cs*	2
85	ab	*S4*	2
85	c	*C4*	2
85	de	*Ci*	2
85	f	*C2*	2
86	ab	*S4*	2
86	cd	*Ci*	2
86	ef	*C2*	2
87	c	*C2h*	2
87	d	*S4*	2
87	f	*Ci*	2
87	g	*C2*	2
88	ab	*S4*	4
88	cd	*Ci*	4
88	e	*C2*	2
89	ef	*D2*	2
89	i	*C2*	2
90	ab	*D2*	2
90	c	*C4*	2
90	d	*C2*	2
91	abc	*C2*	4
92	a	*C2*	4
93	abcdef	*D2*	2
93	ijklmno	*C2*	2

94	ab	*D2*	2
94	cdef	*C2*	2
95	abc	*C2*	4
96	a	*C2*	4
97	cd	*D2*	2
97	f	*C2*	2
98	ab	*D2*	4
98	c	*C2*	2
98	def	*C2*	4
99	c	*C2v*	2
100	a	*C4*	2
100	b	*C2v*	2
101	c	*C2*	2
102	a	*C2v*	2
102	b	*C2*	2
103	c	*C2*	2
104	a	*C4*	2
104	b	*C2*	2
105	c	*C2v*	2
106	ab	*C2*	2
107	b	*C2v*	2
108	b	*C2v*	2
109	a	*C2v*	2
109	b	*Cs*	2
110	a	*C2*	2
111	ef	*D2*	2
111	m	*C2*	2
112	abcd	*D2*	2
112	ef	*S4*	2
112	ghijm	*C2*	2
113	ab	*S4*	2
113	c	*C2v*	2
113	d	*C2*	2
114	ab	*S4*	2
114	cd	*C2*	2
115	g	*C2v*	2
116	ab	*D2*	2
116	cd	*S4*	2
116	efi	*C2*	2
117	ab	*S4*	2
117	cd	*D2*	2
117	ef	*C2*	2
118	ab	*S4*	2

118	cd	*D2*	2
118	efgh	*C2*	2
119	f	*C2v*	2
119	h	*C2*	2
120	ad	*D2*	2
120	bc	*S4*	2
120	eg	*C2*	2
121	c	*D2*	2
121	d	*S4*	2
121	h	*C2*	2
122	ab	*S4*	4
122	c	*C2*	2
122	d	*C2*	4
123	ef	*D2h*	2
123	i	*C2v*	2
124	ac	*D4*	2
124	bd	*C4h*	2
124	e	*C2h*	2
124	f	*D2*	2
124	ijkl	*C2*	2
124	m	*Cs*	2
125	ab	*D4*	2
125	cd	*D2d*	2
125	ef	*C2h*	2
125	g	*C4*	2
125	h	*C2v*	2
125	kl	*C2*	2
126	ab	*D4*	2
126	c	*D2*	2
126	d	*S4*	2
126	e	*C4*	2
126	f	*Ci*	2
126	ghij	*C2*	2
127	ab	*C4h*	2
127	cd	*D2h*	2
127	e	*C4*	2
127	f	*C2v*	2
128	ab	*C4h*	2
128	c	*C2h*	2
128	d	*D2*	2
128	e	*C4*	2
128	fg	*C2*	2
128	h	*Cs*	2

129	ab	*D2d*	2
129	c	*C4v*	2
129	de	*C2h*	2
129	f	*C2v*	2
129	i	*Cs*	2
130	a	*D2*	2
130	b	*S4*	2
130	c	*C4*	2
130	d	*Ci*	2
130	ef	*C2*	2
131	abcd	*D2h*	2
131	ef	*D2d*	2
131	ijklm	*C2v*	2
131	n	*C2*	2
131	q	*Cs*	2
132	ac	*D2h*	2
132	bd	*D2d*	2
132	e	*D2*	2
132	f	*C2h*	2
132	ij	*C2v*	2
132	klm	*C2*	2
132	n	*Cs*	2
133	abc	*D2*	2
133	d	*S4*	2
133	e	*Ci*	2
133	fghij	*C2*	2
134	ab	*D2d*	2
134	cd	*D2*	2
134	ef	*C2h*	2
134	g	*C2v*	2
134	hijkl	*C2*	2
135	ac	*C2h*	2
135	b	*S4*	2
135	d	*D2*	2
135	efg	*C2*	2
135	h	*Cs*	2
136	ab	*D2h*	2
136	c	*C2h*	2
136	d	*S4*	2
136	efg	*C2v*	2
136	h	*C2*	2
136	i	*Cs*	2
137	ab	*D2d*	2

137	cd	*C2v*	2
137	e	*Ci*	2
137	f	*C2*	2
137	g	*Cs*	2
138	a	*D2*	2
138	b	*S4*	2
138	cd	*C2h*	2
138	e	*C2v*	2
138	fgh	*C2*	2
139	c	*D2h*	2
139	d	*D2d*	2
139	f	*C2h*	2
139	g	*C2v*	2
139	k	*C2*	2
140	a	*D4*	2
140	b	*D2d*	2
140	c	*C4h*	2
140	d	*D2h*	2
140	e	*C2h*	2
140	g	*C2v*	2
140	ij	*C2*	2
140	k	*Cs*	2
141	ab	*D2d*	4
141	cd	*C2h*	4
141	e	*C2v*	2
141	fg	*C2*	4
141	h	*Cs*	2
142	a	*S4*	4
142	b	*D2*	4
142	c	*Ci*	4
142	d	*C2*	2
142	ef	*C2*	4
143	\	\	\
144	\	\	\
145	\	\	\
146	\	\	\
147	d	*C3*	3
147	ef	*Ci*	2
148	de	*Ci*	2
149	\	\	\
150	d	*C3*	3
151	ab	*C2*	3
152	ab	*C2*	3

153	ab	*C2*	3
154	ab	*C2*	3
155	\	\	\
156	\	\	\
157	b	*C3*	3
158	\	\	\
159	b	*C3*	3
160	\	\	\
161	\	\	\
162	cd	*D3*	3
162	fg	*C2h*	2
162	h	*C3*	3
163	a	*D3*	2
163	b	*C3i*	2
163	cd	*D3*	2,3
163	f	*C3*	3
163	g	*Ci*	2
163	h	*C2*	2
164	d	*C3v*	3
164	ef	*C2h*	2
165	a	*D3*	2
165	b	*C3i*	2
165	d	*C3*	3
165	e	*Ci*	2
165	f	*C2*	2
166	de	*C2h*	2
167	a	*D3*	2
167	b	*C3i*	2
167	d	*Ci*	2
167	e	*C2*	2
168	b	*C3*	3
168	c	*C2*	2
169	\	\	\
170	\	\	\
171	b	*C2*	2
172	b	*C2*	2
173	b	*C3*	3
174	\	\	\
175	cd	*C3h*	3
175	fg	*C2h*	2
175	h	*C3*	3
175	i	*C2*	2
176	a	*C3h*	2

176	b	*C3i*	2
176	cd	*C3h*	2,3
176	f	*C3*	3
176	g	*Ci*	2
176	h	*Cs*	2
177	cd	*D3*	3
177	fg	*D2*	2
177	h	*C3*	3
177	i	*C2*	2
178	ab	*C2*	6
179	ab	*C2*	6
180	ab	*D2*	3
180	cd	*D2*	2,3
180	f	*C2*	2
180	ghij	*C2*	3
181	ab	*D2*	3
181	cd	*D2*	2,3
181	f	*C2*	2
181	ghij	*C2*	3
182	ab	*D3*	2
182	cd	*D3*	2,3
182	f	*C3*	3
182	gh	*C2*	2
183	b	*C3v*	3
183	c	*C2v*	2
184	b	*C3*	3
184	c	*C2*	2
185	b	*C3*	3
186	b	*C3v*	3
187	\	\	\
188	ace	*D3*	2
188	bdf	*C3h*	2
188	j	*C2*	2
188	k	*Cs*	2
189	cd	*C3h*	3
189	h	*C3*	3
190	a	*D3*	2
190	b	*C3h*	2
190	cd	*C3h*	2,3
190	f	*C3*	3
190	g	*C2*	2
190	h	*Cs*	2
191	cd	*D3h*	3

191	fg	*D2h*	2
191	h	*C3v*	3
191	i	*C2v*	2
192	a	*D6*	2
192	b	*C6h*	2
192	c	*D3*	2,3
192	d	*C3h*	2,3
192	f	*D2*	2
192	g	*C2h*	2
192	h	*C3*	3
192	ijk	*C2*	2
192	l	*Cs*	2
193	a	*D3h*	2
193	b	*D3d*	2
193	c	*C3h*	2,3
193	d	*D3*	2,3
193	f	*C2h*	2
193	g	*C2v*	2
193	h	*C3*	3
193	i	*C2*	2
193	j	*Cs*	2
194	a	*D3d*	2
194	b	*D3h*	2
194	cd	*D3h*	2,3
194	f	*C3v*	3
194	g	*C2h*	2
194	h	*C2v*	2
194	i	*C2*	2
194	j	*Cs*	2
195	cd	*D2*	2
195	gh	*C2*	2
196	g	*C2*	2
197	b	*D2*	2
197	e	*C2*	2
198	\	\	\
199	b	*C2*	4
200	cd	*D2h*	2
200	fg	*C2v*	2
201	a	*T*	2
201	bc	*C3i*	2
201	d	*D2*	2
201	fg	*C2*	2
202	c	*T*	2

202	d	*C2h*	4
202	g	*C2*	2
203	ab	*T*	4
203	cd	*C3i*	4
203	f	*C2*	4
204	b	*D2h*	2
204	c	*C3i*	2
204	e	*C2v*	2
205	ab	*C3i*	2
206	ab	*C3i*	2
206	d	*C2*	4
207	cd	*D4*	2
207	h	*C2*	2
208	a	*T*	2
208	bc	*D3*	2
208	d	*D2*	2
208	ef	*D2*	4
208	hijkl	*C2*	2
209	c	*T*	2
209	d	*D2*	4
209	i	*C2*	2
210	ab	*T*	4
210	cd	*D3*	4
210	fg	*C2*	4
211	b	*D4*	2
211	c	*D3*	2
211	d	*D2*	4
211	gi	*C2*	2
212	ab	*D3*	4
212	d	*C2*	4
213	ab	*D3*	4
213	d	*C2*	4
214	ab	*D3*	4
214	cd	*D2*	8
214	fgh	*C2*	4
215	cd	*D2d*	2
215	h	*C2*	2
216	g	*C2v*	2
217	b	*D2d*	2
217	d	*S4*	4
217	f	*C2*	2
218	a	*T*	2
218	b	*D2*	2

218	cd	*S4*	4
218	fgh	*C2*	2
219	ab	*T*	2
219	cd	*S4*	4
219	fg	*C2*	2
220	ab	*S4*	8
220	d	*C2*	4
221	cd	*D4h*	2
221	h	*C2v*	2
222	a	*O*	2
222	b	*D4*	2
222	c	*C3i*	2
222	d	*S4*	4
222	e	*C4*	2
222	gh	*C2*	2
223	a	*Th*	2
223	b	*D2h*	2
223	cd	*D2d*	4
223	e	*D3*	2
223	fgh	*C2v*	2
223	j	*C2*	2
223	k	*Cs*	2
224	a	*Td*	2
224	bc	*D3d*	2
224	d	*D2d*	2
224	f	*D2*	4
224	g	*C2v*	2
224	hij	*C2*	2
225	c	*Td*	2
225	d	*D2h*	4
225	g	*C2v*	2
226	a	*O*	2
226	b	*Th*	2
226	c	*D2d*	4
226	d	*C4h*	4
226	e	*C2v*	2
226	f	*C4*	2
226	h	*C2*	2
226	i	*Cs*	2
227	ab	*Td*	4
227	cd	*D3d*	4
227	f	*C2v*	4
227	h	*C2*	4

228	a	*T*	4
228	b	*D3*	4
228	c	*C3i*	4
228	d	*S4*	4
228	fg	*C2*	4
229	b	*D4h*	2
229	c	*D3d*	2
229	d	*D2d*	4
229	g	*C2v*	2
229	i	*C2*	2
230	a	*C3i*	4
230	b	*D3*	4
230	c	*D2*	8
230	d	*S4*	8
230	fg	*C2*	4

2. The FQFE in Sc_2CO_2

In this section, we first constructed the ferroelectric phase L_2 from a given L_1 phase of monolayer Sc_2CO_2. Then we discuss the FQFE contributed by several atoms.

L_2 is constructed by 1) Dividing L_1 (See Fig. S1(a)) into two parts, M (two O atoms) and F (two Sc atoms and one C atom, see Fig. S1(b)). In this way, G_F (=*P-3m1*) is a supergroup of G_L (=*P3m1*). Besides the symmetries in G_L, F has the inversion symmetry I, i.e. $G_F=G_L+IG_L$=*P-3m1* (No. 164). The inversion center is in the middle point of two Sc atoms. 2) Apply inversion to L_1, we get L_2 (see Fig. S1(c)).

The top and bottom O atom layers is dubbed as M^1 and M^2, respectively. The structure consisting of M^i and F can be viewed as a ferroelectric phase of the simple FQFE case. The Wyckoff position $\mathbf{M}^1$ (0,0,-z) and $\mathbf{M}^2$ (1/3,2/3,z) in G_F (see Tab. S2) are 2c and 2d, respectively. The inversion operation will turn $\mathbf{M}^1$(0,0,-z) and $\mathbf{M}^2$(1/3,2/3,z) into $\mathbf{M}^{1'}$(0,0,z) and $\mathbf{M}^{2'}$(2/3,1/3,-z). Since the top and bottom layers are exchanged, the fractional polarization contributed by the two atoms are $\Delta\mathbf{P}^1=\mathbf{M}^{2'}-\mathbf{M}^1$=(2/3,1/3,0) and $\Delta\mathbf{P}^2=\mathbf{M}^{1'}-\mathbf{M}^2$=(-1/3,-2/3,0). Obviously, $\Delta\mathbf{P}^1$ is equivalent to $\Delta\mathbf{P}^2$. If $\mathbf{Q}$ is set as the polarization quantum along the [210] direction, $\Delta\mathbf{P}^1=\Delta\mathbf{P}^2=\frac{1}{3}\mathbf{Q}$ and the total fractionally quantized polarization $\Delta\mathbf{P}=\frac{2}{3}\mathbf{Q}$.

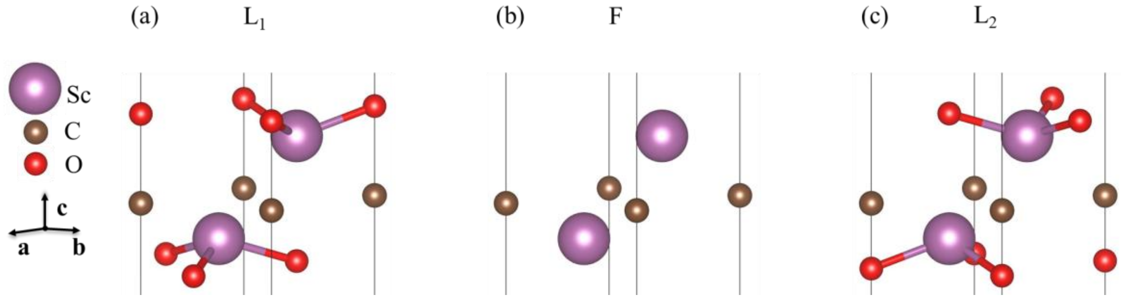

Figure S1. Structure of monolayer Sc_2CO_2. Side views of monolayer Sc_2CO_2, corresponding to (a) L_1 phase (No. 156 space group *P3m1*, point group C_{3v}), (b) structure F (No. 164 space group *P-3m1*, point group D_{3d}), and (c) L_2 phases (No. 156 space group *P3m1*, point group C_{3v}), respectively.

3. The Wyckoff Position Tables of *P-3m1* and *Fm-3m*

In the case of the monolayer α-In_2Se_3 and Sc_2CO_2, G_F is *P-3m1* and the Wyckoff position table is shown in Table S2. For AgBr, G_F is *Fm-3m* and the Wyckoff position table is shown in Tab. S3.

Table. S2. Wyckoff position table [1] of No.164 space group, *P-3m1*. In α-In_2Se_3, M is the middle Se atom and its Wyckoff position in G_F=*P-3m1* is 2d.

Multiplicity	Wyckoff letter	Site symmetry	Coordinates
12	j	C_1	(x,y,z) (-y,x-y,z) (-x+y,-x,z) (y,x,-z) (x-y,-y,-z) (-x,-x+y,-z) (-x,-y,-z) (y,-x+y,-z) (x-y,x,-z) (-y,-x,z) (-x+y,y,z) (x,x-y,z)
6	i	C_s	(x,-x,z) (x,2x,z) (-2x,-x,z) (-x,x,-z) (2x,x,-z) (-x,-2x,-z)
6	h	C_2	(x,0,1/2) (0,x,1/2) (-x,-x,1/2) (-x,0,1/2) (0,-x,1/2) (x,x,1/2)
6	g	C_2	(x,0,0) (0,x,0) (-x,-x,0) (-x,0,0) (0,-x,0) (x,x,0)
3	f	C_{2h}	(1/2,0,1/2) (0,1/2,1/2) (1/2,1/2,1/2)
3	e	C_{2h}	(1/2,0,0) (0,1/2,0) (1/2,1/2,0)
2	d	C_{3v}	(1/3,2/3,z) (2/3,1/3,-z)
2	c	C_{3v}	(0,0,z) (0,0,-z)
1	b	D_{3d}	(0,0,1/2)
1	a	D_{3d}	(0,0,0)

Table. S3. Wyckoff position table [1] of No.225 space group, *Fm-3m* (G_F). In AgBr, F locates at 4a and M moves from a coordinate in 8c to 4b then to another coordinate in 8c.

Multiplicity	Wyckoff letter	Site symmetry	Coordinates (0,0,0) + (0,1/2,1/2) + (1/2,0,1/2) + (1/2,1/2,0) +			
192	l	C_1	(x,y,z)	(-x,-y,z)	(-x,y,-z)	(x,-y,-z)
			(z,x,y)	(z,-x,-y)	(-z,-x,y)	(-z,x,-y)
			(y,z,x)	(-y,z,-x)	(y,-z,-x)	(-y,-z,x)
			(y,x,-z)	(-y,-x,-z)	(y,-x,z)	(-y,x,z)
			(x,z,-y)	(-x,z,y)	(-x,-z,-y)	(x,-z,y)
			(z,y,-x)	(z,-y,x)	(-z,y,x)	(-z,-y,-x)
			(-x,-y,-z)	(x,y,-z)	(x,-y,z)	(-x,y,z)
			(-z,-x,-y)	(-z,x,y)	(z,x,-y)	(z,-x,y)
			(-y,-z,-x)	(y,-z,x)	(-y,z,x)	(y,z,-x)
			(-y,-x,z)	(y,x,z)	(-y,x,-z)	(y,-x,-z)
			(-x,-z,y)	(x,-z,-y)	(x,z,y)	(-x,z,-y)
			(-z,-y,x)	(-z,y,-x)	(z,-y,-x)	(z,y,x)
96	k	C_s	(x,x,z)	(-x,-x,z)	(-x,x,-z)	(x,-x,-z)
			(z,x,x)	(z,-x,-x)	(-z,-x,x)	(-z,x,-x)
			(x,z,x)	(-x,z,-x)	(x,-z,-x)	(-x,-z,x)
			(x,x,-z)	(-x,-x,-z)	(x,-x,z)	(-x,x,z)
			(x,z,-x)	(-x,z,x)	(-x,-z,-x)	(x,-z,x)
			(z,x,-x)	(z,-x,x)	(-z,x,x)	(-z,-x,-x)
96	j	C_s	(0,y,z)	(0,-y,z)	(0,y,-z)	(0,-y,-z)
			(z,0,y)	(z,0,-y)	(-z,0,y)	(-z,0,-y)
			(y,z,0)	(-y,z,0)	(y,-z,0)	(-y,-z,0)
			(y,0,-z)	(-y,0,-z)	(y,0,z)	(-y,0,z)

			(0,*z*,-*y*) (0,*z*,*y*) (0,-*z*,-*y*) (0,-*z*,*y*)<br>(*z*,*y*,0) (*z*,-*y*,0) (-*z*,*y*,0) (-*z*,-*y*,0)
**48**	i	C_{2v}	(1/2,*y*,*y*) (1/2,-*y*,*y*) (1/2,*y*,-*y*) (1/2,-*y*,-*y*)<br>(*y*,1/2,*y*) (*y*,1/2,-*y*) (-*y*,1/2,*y*) (-*y*,1/2,-*y*)<br>(*y*,*y*,1/2) (-*y*,*y*,1/2) (*y*,-*y*,1/2) (-*y*,-*y*,1/2)
**48**	h	C_{2v}	(0,*y*,*y*) (0,-*y*,*y*) (0,*y*,-*y*) (0,-*y*,-*y*)<br>(*y*,0,*y*) (*y*,0,-*y*) (-*y*,0,*y*) (-*y*,0,-*y*)<br>(*y*,*y*,0) (-*y*,*y*,0) (*y*,-*y*,0) (-*y*,-*y*,0)
**48**	g	C_{2v}	(*x*,1/4,1/4) (-*x*,3/4,1/4) (1/4,*x*,1/4) (1/4,-*x*,3/4)<br>(1/4,1/4,*x*) (3/4,1/4,-*x*) (1/4,*x*,3/4) (3/4,-*x*,3/4)<br>(*x*,1/4,3/4) (-*x*,1/4,1/4) (1/4,1/4,-*x*) (1/4,3/4,*x*)
**32**	f	C_{3v}	(*x*,*x*,*x*) (-*x*,-*x*,*x*) (-*x*,*x*,-*x*) (*x*,-*x*,-*x*)<br>(*x*,*x*,-*x*) (-*x*,-*x*,-*x*) (*x*,-*x*,*x*) (-*x*,*x*,*x*)
**24**	e	C_{4v}	(*x*,0,0) (-*x*,0,0) (0,*x*,0) (0,-*x*,0)<br>(0,0,*x*) (0,0,-*x*)
**24**	d	D_{2h}	(0,1/4,1/4) (0,3/4,1/4) (1/4,0,1/4) (1/4,0,3/4)<br>(1/4,1/4,0) (3/4,1/4,0)
**8**	c	T_d	(1/4,1/4,1/4) (1/4,1/4,3/4)
**4**	b	O_h	(1/2,1/2,1/2)
**4**	a	O_h	(0,0,0)

4. Materials with both *F-43m* and *Fm-3m* phases

We conducted a screening in the Materials Project database for materials exhibiting both *F-43m* and *Fm-3m* phases, and the corresponding results are presented in Table S4.

Table. S4. Energy differences between the H phases (rock salt) and L phase (zinc blende), $\Delta E = E(\mathrm{H}) - E(\mathrm{L})$.

Formula	ΔE (meV/f.u.)	Formula	ΔE (meV/f.u.)
BeS	1118	ZnO	276
BeSe	1087	ZnS	636
BeTe	1019	ZnSe	614
BN	3506	GaP	811
BP	2130	CdS	269
BAs	1776	CdSe	293
AlP	583	CdTe	359
AlSb	427	AgBr	74
AlAs	558	AgI	190
SiC	1480	InP	492

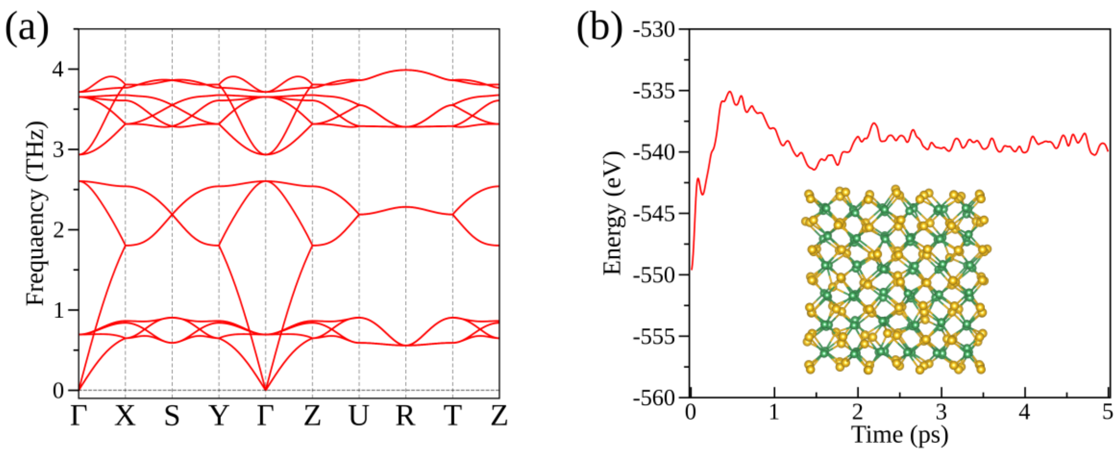

Figure S2. Dynamical and thermal stability of AgBr. (a) Phonon spectrum along the high-symmetry path Γ → X → S → Y → Γ → Z → U → R → T → Z of AgBr for *F-43m* phase. (b) *Ab initio* molecular dynamics simulations of AgBr for *F-43m* phase at 300 K. The inset shows the corresponding structure after 5 ps of simulation.

5. Ferroelasticity of non-polar FQFE

In the ferroelectric phase transition of a system with a square H phase (see Fig. 1(c) in the main text), the low symmetry phases L_1 and L_2 do not have the four-fold rotational symmetry. Thus, there should be lattice distortion in the two phases (see Fig. S3) and in this case the FQFE can be ferroelastic.

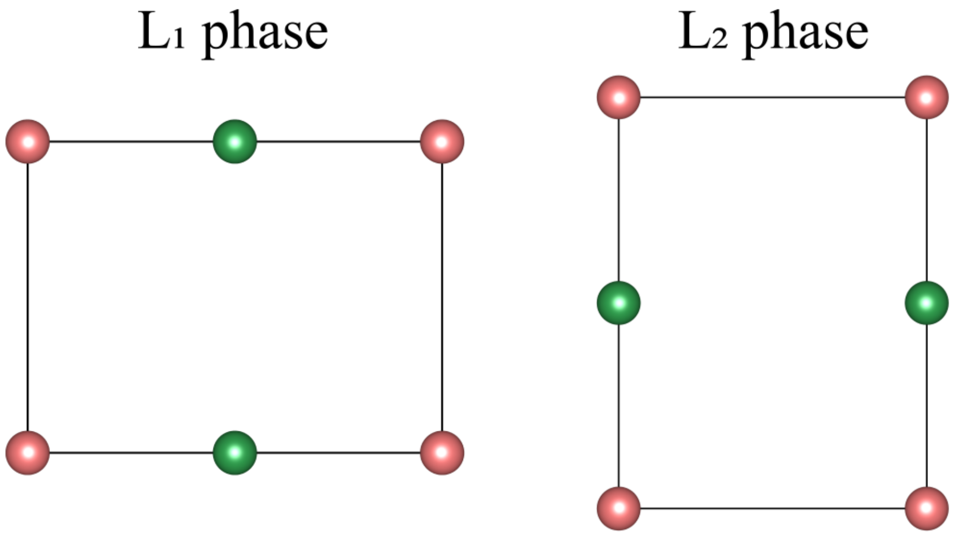

Figure S3. Ferroelasticity of non-polar FQFE, corresponding to the phase transition of Fig. 1(c) in the main text.

6. The thermal-stability analysis of FQFE materials

We perform ab initio molecular dynamics (AIMD) simulations for the FQFE systems of monolayer In_2Se_3 and bulk AgBr, for both the FE state and the intermediate state. As shown in Fig. S4, both example FQFE systems exhibit good thermal-stability at room-temperature.

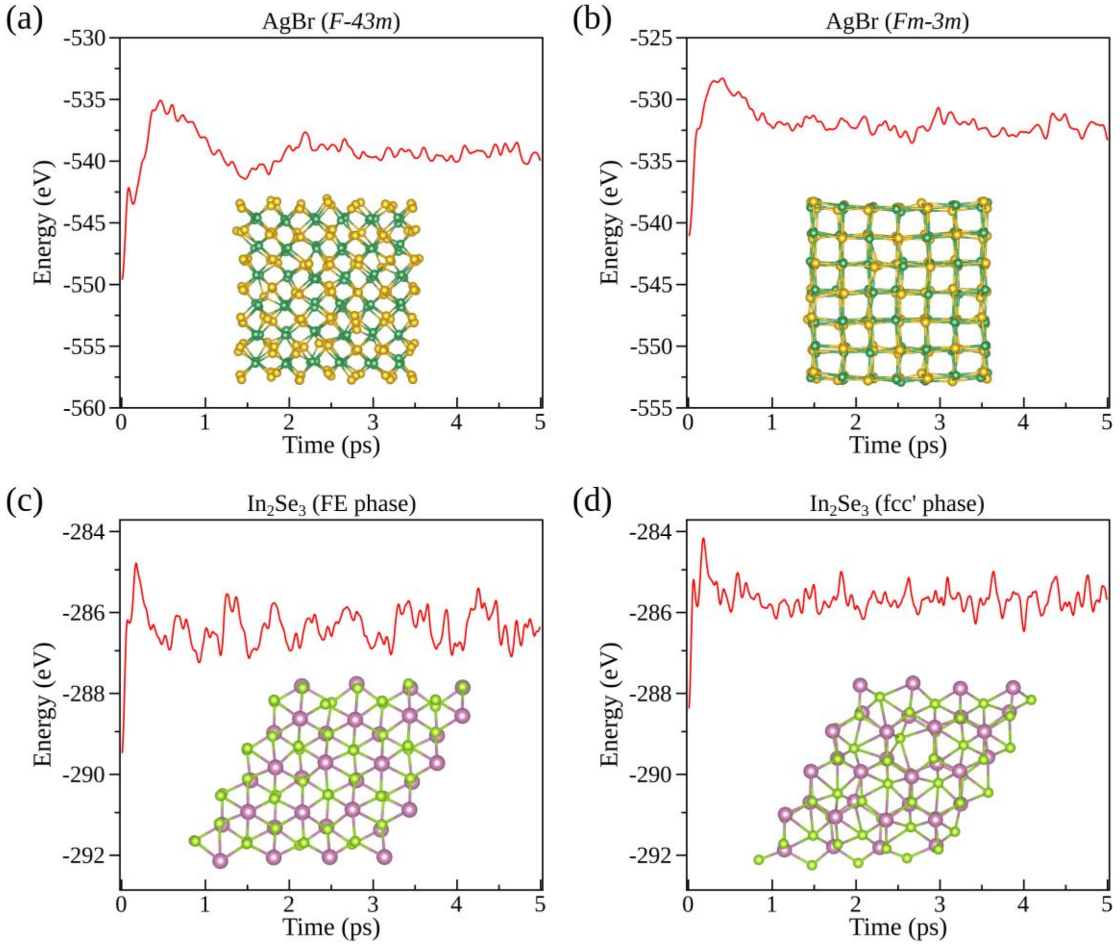

Fig. S4 Thermal stability of FQFE materials. (a) and (b) are the ab initio molecular dynamics (AIMD) simulations of AgBr for *F-43m* and *Fm-3m* phase (L and H phase) at 300 K. (c) and (d) are the AIMD simulations of monolayer α-In_2Se_3 for FE and fcc' phase at 300 K. The inset shows the corresponding structure after 5 ps of simulation.

7. FQFE in organic-inorganic materials

The structure of NH_4Br is constructed by replacing the Ag atom of AgBr by NH_4^+. The fractional polarization is similar to that in AgBr. This can also be considered as an instance of FQFE with multi-atom movements.

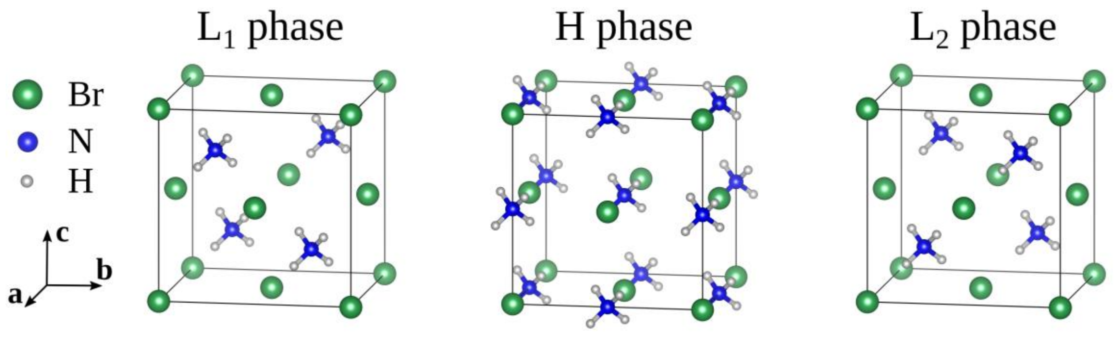

Fig. S5 Illustration of realizing FQFE in molecular material of NH_4Br, where the NH_4^+ takes the position of Ag in AgBr.

8. Energy barrier and polarization difference along different switching paths for monolayer α-In_2Se_3.

Nudged elastic band (NEB) calculations of the energy barrier are performed and evolution of the polarization is obtained using Berry phase method using different reference structures, which includes the PE phase, H phase, and their respective optimized phase transition paths [see Fig. S6]. One can find that the phase in fcc' [2] yields the most energetically favorable switching path with the energy barrier of 68 meV/f.u. Moreover, given the initial and final states, the polarization difference $\Delta\mathbf{P} = \mathbf{P}_1 - \mathbf{P}_2$ can only differ by an integer quantum polarization for different switching paths [see Fig. S7].

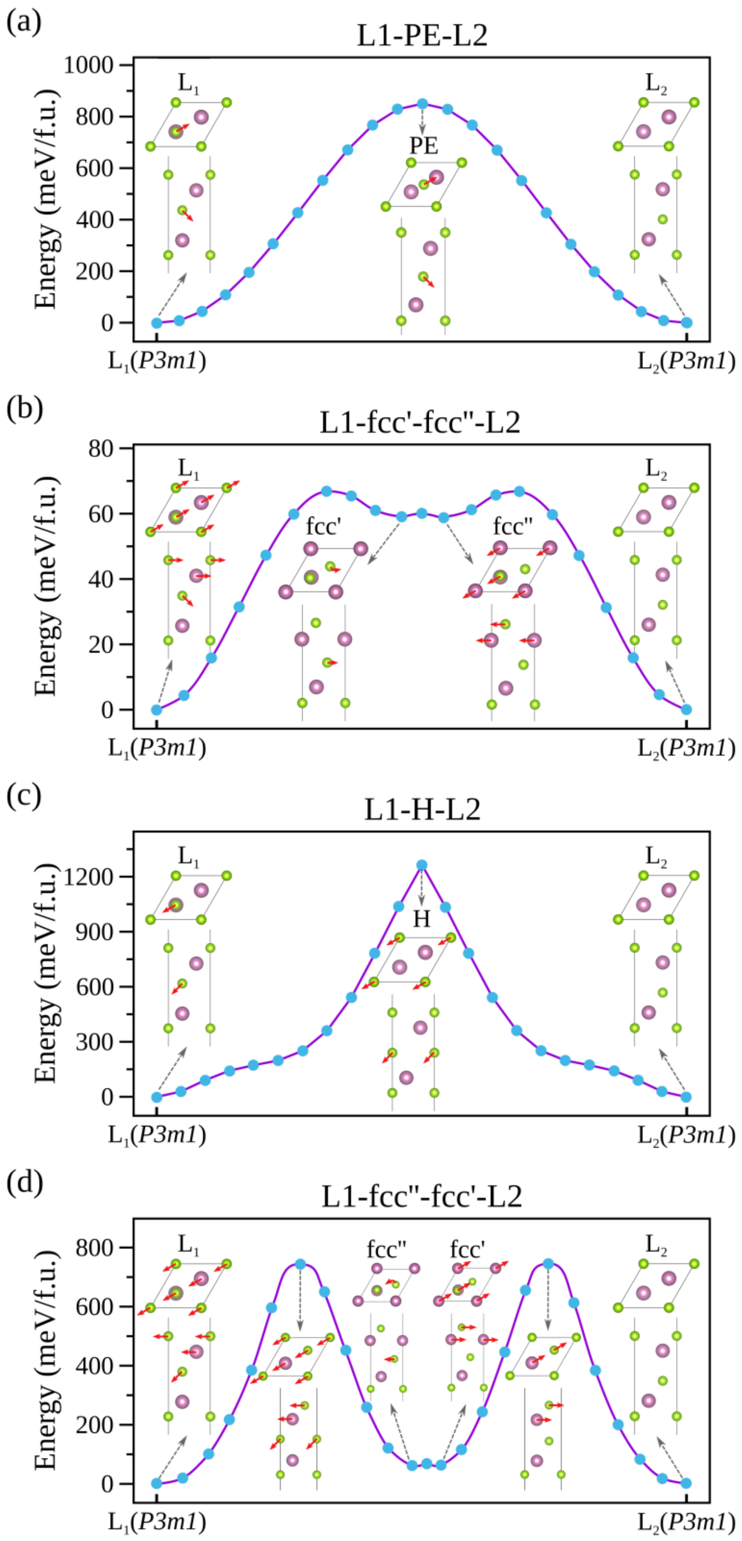

Fig. S6 NEB calculation of the energy barrier for monolayer α-In_2Se_3 along switching paths with intermediate state (a) PE phase (b) fcc' phase (c) H phase (d) fcc'' phase. The energy barriers are 850 meV/f.u., 68 meV/f.u., 1262 meV/f.u. and 625 meV/f.u. for (a), (b), (c), (d), respectively. The red arrows represent the movements of ions during the switching.

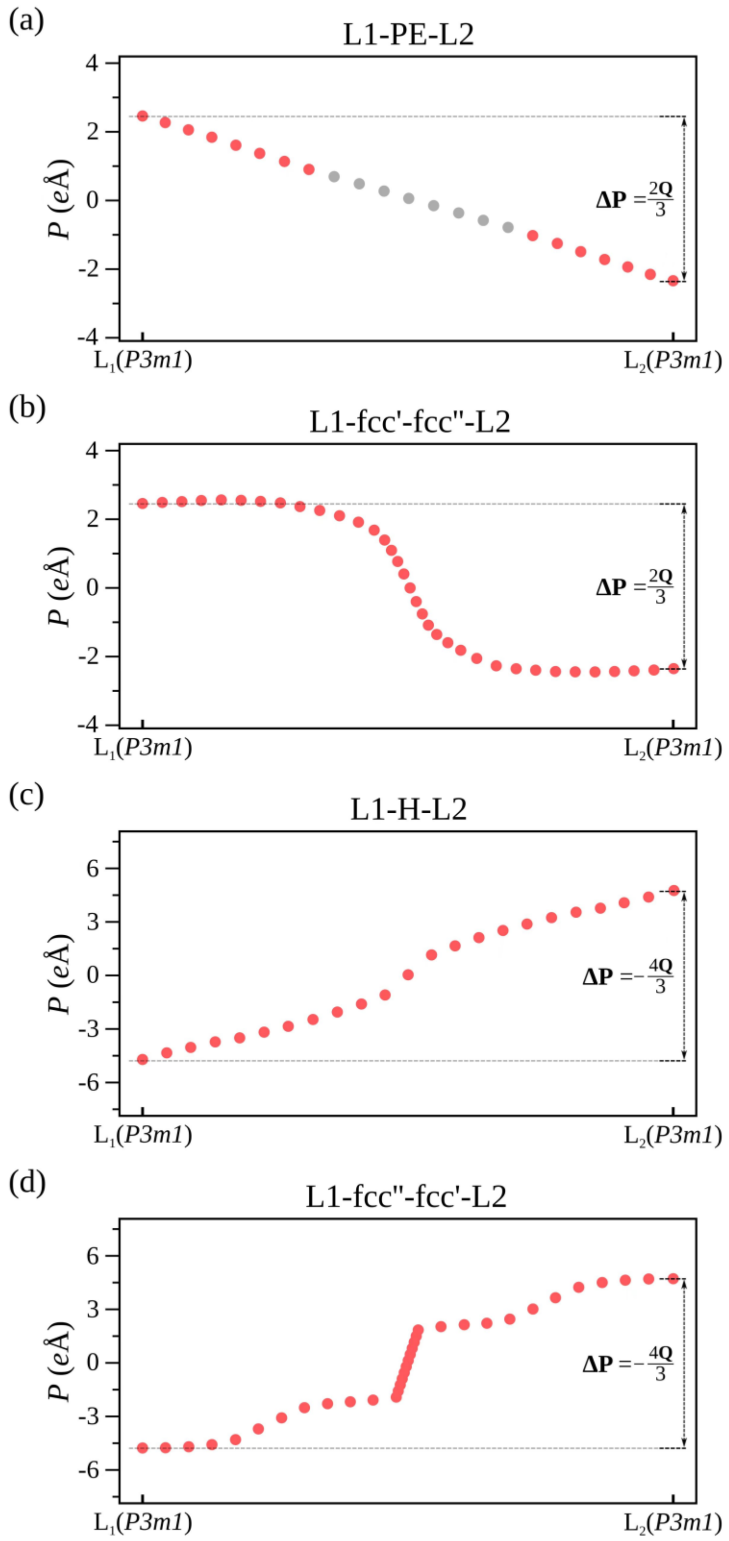

Fig. S7 The evolution of the polarization for monolayer α-In_2Se_3 in the primitive cell along the paths in Fig. S6: (a) PE phase (b) fcc' phase (c) H phase (d) fcc'' phase. **Q** is the polarization quantum along the [110] direction. The polarization difference $\Delta\mathbf{P}=\mathbf{P}_1-\mathbf{P}_2$ are 4.74 *e*Å, 4.74 *e*Å, -9.48 *e*Å and -9.48 *e*Å per unit cell for (a), (b), (c), (d), respectively. The polarization of the gray points in panel (a) is determined by fitting other structures in this path due to their metallic behavior.

References

1. Aroyo MI*, et al.* Bilbao Crystallographic Server: I. Databases and crystallographic computing programs. *Z. Krist. - Cryst. Mater.* **221**, 15-27 (2006).

2. Ding W*, et al.* Prediction of intrinsic two-dimensional ferroelectrics in In2Se3 and other III2-VI3 van der Waals materials. *Nat. Commun.* **8**, 14956 (2017).
